# State of the art in anti-cancer mAbs

**DOI:** 10.1186/s12929-016-0311-y

**Published:** 2017-02-20

**Authors:** S. M. Chiavenna, J. P. Jaworski, A. Vendrell

**Affiliations:** 1 Ferrer Advanced Biotherapeutics, Ferrer Internacional, Barcelona, Spain; 2 Institute of Virology, CICVyA, INTA - CONICET, Castelar, Buenos Aires, Argentina; 3 Faculty of Medicine, CEFYBO – CONICET/University of Buenos Aires, C.A.B.A., Argentina

**Keywords:** Cancer, Solid tumors, Immunotherapy, Monoclonal antibody, EGFR, HER2, VEGF/VEGFR, CTLA-4, PD-1/PD-L1, RANK/RANKL

## Abstract

Following Milstein’s discovery, the monoclonal antibodies (mAbs) became a basic tool for biomedical science. In cancer field, since the first mAb was approved by the FDA a great improvement took place making of them a therapeutic option for many cancer types in the current clinical practice. Today, mAbs are being developed to target different molecules with different mechanisms of action and its target potential is unlimited. However, this huge and fast growing new field needs to be organized to better understand the treatment options we have to confront different cancer diseases. Current cancer targeted immunotherapies aim to achieve different goals like the regulation of osteoclast function, the delivery of cytotoxic drugs into tumor cells and the blockade of oncogenic pathways, neo-angiogenesis and immune checkpoints. Here, we reviewed the most relevant therapeutic mAbs for solid tumors available in current clinical practice.

## Background

Cancer is a leading cause of deaths all over the world and its occurrence is increasing because of the growth and aging of the population, as well as an increasing prevalence of established risk factors [1]. Among therapeutic approaches, surgery, chemotherapy and irradiation keep being the standard of care based on tumor type and stage. However, the chemotherapy success is limited due to lack of selectivity for tumor cells, resulting in systemic toxicity and the appearance of drug-resistant [2]. As we learn more about the molecular characteristics of tumor cells we are able to design better target therapies to block their growths and spread. Most of these therapies are based on small-molecule drugs that easily enter the tumor cells or monoclonal antibodies (mAbs) that binds to specific targets on their surface.

Targeted therapies based on mAbs are immunotherapies aiming different goals such as the blockade of oncogenic pathways with subsequent effects on cell growth and apoptosis [3], the blockade of the formation of new blood vessels [4], the modulation of immune response against tumor cells [5], the regulation of osteoclast function [6] or the delivery of cytotoxic drugs to kill tumor cells [7]. Since the approval of the first mAb (Rituximab®) by the United States Food and Drug Administration (FDA), hundreds of them, including murine, chimeric and humanized, have been developed for cancer treatment [8]. Some of these mAbs have been approved by the FDA and are already available for clinical use in everyday practice, as monotherapy or in combination with standard chemotherapy regimens while many others are still being tested in different clinical trial. A brief description of the most used mAbs in clinical practice is summarized in Table 1 and discussed ahead.

**Table 1:** Summary of the FDA approved monoclonal antibodies for treatment of solid tumors

mAB	Name	Type	Target	FDA approved^a^
Trastuzumab	Herceptin®	Humanized	HER2	HER2-positive metastatic/non-metastatic breast cancer HER2-positive metastatic gastric or gastroesophageal junction adenocarcinoma
Pertuzumab	Perjeta®	Humanized	HER2	HER2-positive metastatic breast cancer HER2-positive, locally advanced, inflammatory, or early stage breast cancer
Cetuximab	Erbitux®	Chimeric	EGFR	Metastatic CRC HNSCC
Panitumumab	Vectibix®	Human	EGFR	Metastatic CRC
Necitumumab	Portrazza™	Human	EGFR	Metastatic squamous NSCLC
Dinutuximab	Unituxin™	Chimeric	GD2	Pediatric high risk neuroblastoma
Bevacizumab	Avastin®	Humanized	VEGF-A	Metastatic CRC Recurrent or metastatic non-squamous NSCLC HER2-negative metastatic breast cancer (revoked in 2011) Metastatic renal cell carcinoma Persistent, recurrent or metastatic cervical cancer Glioblastoma Recurrent epithelial ovarian, fallopian tube, or primary peritoneal cancer
Ramucirumab	Ciramza®	Human	VEGFR-2	Advanced or metastatic gastric or gastroesophageal junction adenocarcinoma Metastatic NSCLC Metastatic CRC
Olaratumab	Lartruvo®	Human	PDGFR-α	Soft tissue sarcoma
Ipilimumab	Yervoy®	Human	CTLA-4	Unresectable or metastatic melanoma Cutaneous melanoma
Nivolumab	Opdivo®	Human	PD-1	Unresectable or metastatic melanoma Metastatic squamous NSCLC Metastatic NSCLC Advanced RCC Recurrent or metastatic HNSCC
Pembrolizumab	Keytruda®	Humanized	PD-1	Unresectable or metastatic melanoma Metastatic NSCLC Recurrent or metastatic HNSCC
Atezolizumab	Tecentriq™	Humanized	PD-L1	Locally advanced or metastatic urothelial carcinoma Metastatic NSCLC
Ado-trastuzumab emtansine^b^	Kadcyla®	Humanized	HER2	HER2-positive metastatic breast cancer
Denosumab	Xgeva®	Human	RANKL	Bone metastases from solid tumors

*CRC* colorectal cancer, *HNSCC* head & neck squamous cell carcinoma, *NSCLC* non-small cell lung cancer, *RCC* renal cell carcinoma

^a^See the text for further details

^b^Trastuzumab covalently linked to emtansine (DM1)

## Blockade of oncogenic pathways

Carcinogenesis usually begins with spontaneous undesired mutations that over activate proto-oncogenes and/or inactivate tumor suppressor genes. During this process a number of cell growth factors or enzymes implicated in the proliferation process become overexpressed. These molecules involved in different oncogenic pathways are known as tumor associated antigens (TAA) and represent optimal blocking targets to avoid the cancer cell proliferation [9]. Thus, mAbs directed to these oncogenic pathways not only can stimulate antibody-dependent cellular cytotoxicity (ADCC) and complement-dependent cytotoxicity (CDC) but also have an intrinsic anticancer activity on the tumor cell [10]. One example of this is the Epidermal Growth Factor Receptor (EGFR) family. The EGFR family is a group of cell surface receptors implicated in the growth of normal epidermal tissue. EGFR and/or its ligands have been shown to be overexpressed in several tumors of epithelial origin.

### EGFR (also erbb1, HER1)

EGFR is perhaps the most widely studied member of the EGFR family and extensive research provides compelling evidence for using EGFR as a target for anticancer therapy. Abnormal receptor activation or dysregulation of the EGFR signal transduction pathways can result from a number of different mechanisms that are potentially relevant to the growth and/or development of human carcinomas [11]. The resulting signaling output from activated EGFR is highly dependent on the activating ligand, as well as the cellular levels of other co-receptors like EGFR members [12]. Furthermore, EGFR acts as a point of integration for signals arising from G-protein–coupled receptors and cytokine receptors, thus it can cross-talk with various heterologous receptors activated by neurotransmitters, lymphokines and stress inducers [12].

EGFR represents today one of the most important targets for cancer therapy, especially for the treatment of metastatic colorectal cancer (mCRC) and head and neck cancers. Some of the mAbs developed to target this pathway are described below.

#### Cetuximab (Erbitux®)

Cetuximab is a recombinant, human/mouse chimeric monoclonal antibody that binds specifically to the extracellular domain of the human EGFR [13, 14]. This binding inhibits the activation of the tyrosine kinase receptor [15] and the associated downstream signaling resulting in inhibition of cell growth, induction of apoptosis and decreased matrix metalloproteinase and vascular endothelial growth factor production [13]. In addition, Cetuximab promotes the receptor dimerization, internalization and degradation leading to receptor down-regulation [16]. Moreover, it can also mediate ADCC, contributing to its antitumoral effect [17]. As signal transduction through the EGFR results in activation of wild-type K-Ras protein, tumor cells with activating K-Ras somatic mutations are continuously active and appears to be independent of EGFR regulation [13].

Erbitux® was first approved by the FDA in 2004 for the treatment of EGFR-expressing mCRC under different circumstances: (i) in combination with FOLFIRI (irinotecan, folinic acid and 5-fluorouracil) as a first-line treatment, (ii) in combination with irinotecan in patients who are refractory to irinotecan-based chemotherapy and (iii) as a single agent in patients who have failed oxaliplatin and irinotecan based chemotherapy or who are intolerant to irinotecan. However, retrospective analyses showed no treatment benefit for Erbitux in patients whose tumors had KRAS mutations in codon 12 or 13. Since then, it is not indicated for treatment of Ras-mutant colorectal cancer or when its status is unknown [13, 18].

Later on, the FDA approved Erbitux® for the treatment of squamous cell carcinoma of the head and neck in combination with radiation therapy for locoregional advanced disease, in combination with platinum-based therapy and 5-FU for recurrent locoregional or metastatic disease [13, 19] and as a single agent in patients with recurrent or metastatic disease after platinum based therapy failure [13, 20].

#### Panitumumab (Vectibix®)

Panitumumab is a recombinant human mAb that binds specifically to the extracellular domain of EGFR on both normal and tumor cells, and competitively inhibits the binding of ligands for EGFR [21, 22]. Non-clinical studies showed that binding of panitumumab to the EGFR prevents ligand-induced receptor autophosphorylation and activation of receptor-associated kinases which results in the inhibition of cell growth, induction of apoptosis, decreased proinflammatory cytokine and vascular growth factor production, and internalization of the EGFR [21]. Here again, tumor cells with activating K-Ras somatic mutations are continuously active and appears to be independent of EGFR regulation [21].

Vectibix® was first approved by the FDA in 2006 and it is currently indicated as a single agent for the treatment of EGFR-expressing, K-ras wild-type mCRC with disease progression or following fluoropyrimidine-, oxaliplatin- and irinotecan-containing chemotherapy regimens [21]. A retrospective analysis conducted on the samples of the PRIME trial demonstrated that other KRAS mutations in exons 3 and 4 in addition to the well-known exon 2 mutations and NRAS mutations on exons 2, 3 and 4 can determine resistance to panitumumab. Importantly, the use of this mAb was associated with a detrimental effect in mutated patients [23]. For these reasons, the limitation of the use of panitumumab in KRAS and NRAS wild-type patients has been extended also to the use of cetuximab.

#### Necitumumab (Portrazza™)

Necitumumab is a recombinant human IgG1 monoclonal antibody that binds to EGFR and blocks the binding with its ligands. In vitro studies showed that necitumumab induces EGFR internalization and degradation and also led to ADCC in EGFR-expressing cells.

Portrazza™ was approved by the FDA in 2015 as an epidermal growth factor receptor antagonist for first-line treatment of patients with metastatic squamous non-small cell lung cancer in combination with gemcitabine and cisplatin [24].

### HER2 (also erbb-2, neu)

HER2 is a transmembrane tyrosine kinase receptor that belongs to EGFR family. Although HER2 has no known activating ligands, it may be activated by the formation of homodimers and/or heterodimers with any member of the EGFR family which are ligand activated like HER1, HER 3 or HER4 [25–27]. From the ones mentioned above, HER2/HER3 is probably the most active and tumor promoting combination [28–30]. HER2 overexpression may lead to transphoshorylation and activation of downstream signaling pathways including Ras-Raf-MAPK and PI3K-AKT which are involved in the inhibition of apoptosis and promotion of proliferation [31]. As its overexpression is associated with poor prognosis in breast, gastric and esophageal cancer [32–35]. HER2 receptor is an ideal target for cancer treatment.

#### Trastuzumab (Herceptin®)

Trastuzumab is a humanized IgG1 kappa mAb that binds with high affinity to the extracellular domain of HER2 receptor. The mechanisms underlying the therapeutic effect is not completely understood but there is a general consensus that it results from the co-influence of multiple actions like i) inhibiting the ligand-independent HER2/HER3 heterodimerization, ii) preventing the proteolytic cleavage of the HER2 extracellular domain and consequently the formation of the constitutively active p95HER2 fragment and iii) inducing the ADCC toward HER2-positive tumours [25, 26, 33, 36–38].

Herceptin® was first approved by the FDA in 1998 and it is currently indicated for adjuvant treatment of HER2-overexpressing node positive or node negative breast cancer as part of different treatment regimens that included: (i) with doxorubicin, cyclophosphamide and either paclitaxel or docetaxel, (ii) with docetaxel and carboplatin and (iii) as a single agent following multi-modality anthracycline based therapy. Herceptin® is also indicated as first-line treatment of (i) HER2-overexpressing metastatic breast cancer, in combination with paclitaxel and (ii) HER2-overexpressing metastatic gastric and gastroesophageal junction adenocarcinoma, in combination with cisplatin and capecitabine or 5-fluorouracil [39], in those patients who have not received prior treatment for metastatic disease.

#### Pertuzumab (Perjeta®)

Pertuzumab is the first antibody from a novel therapeutic class called dimerization inhibitors. It is a recombinant humanized mAb that binds the extracellular dimerization domain II of HER2, targeting a different epitope to that of trastuzumab [40]. In fact, trastuzumab and pertuzumab work with a complementary action. The first inhibits ligand-independent HER2 signaling without preventing ligand-activated HER2/HER3 or HER2/HER1 heterodimerization, whereas the second prevents the formation of the ligand-induced heterodimers of HER2 with any other member of the EGFRs family. Pertuzumab also mediates ADCC in a similar way to trastuzumab [41, 42] and showed promising antitumor efficacy in vitro and in vivo against several tumor types such as breast cancer, lung cancer, prostate cancer, colorectal cancer and ovarian cancer [43–47].

Perjeta® was first approved by the FDA in 2012 to be used as first-line treatment, in combination with trastuzumab and docetaxel, in patients with HER2-overexpressing metastatic breast cancer and have not received prior anti-HER2 therapy or chemotherapy for metastatic disease. This was done based on the results from the CLEOPATRA study [48]. In 2013, the FDA approved its use in combination with trastuzumab and docetaxel as neoadjuvant treatment of patients with HER2-overexpressing, locally advanced, inflammatory and early stage breast cancer. However, this last indication was only based on pathological complete response (pCR) rate improvements but not event-free survival or overall survival [49, 50].

### Glycolipid disialoganglioside (GD2)

Tumor-associated gangliosides emerged as promising targets for the development of monoclonal antibodies to treat various cancers types. They are glycosylated lipid molecules that belong to the glycosphingolipid class where GD2 stands out as it is over expressed on the cell surface of neuroblastomas but not on the surface of neurons, skin melanocytes and peripheral sensory nerve fibers [51–53]. GD2 can induce phosphorylation of focal adhesion kinase FAK and Lyn kinase increasing cell migration, invasion and motility and its interaction with integrins in glycolipid-enriched microdomain (GEM) raft is likely to be important to control the malignant potential of neuroblastoma [51]. Besides, GD2 was found to be expressed in melanomas, small-cell lung cancer and bone and soft-tissue sarcomas [52–54].

#### Dinutuximab (Unituxin™)

Dinutuximab is a human-murine, anti-GD2 monoclonal antibody that binds to GD2 and induces cell lysis of GD2-expressing cells through antibody-dependent cell-mediated cytotoxicity and complement-dependent cytotoxicity [55, 56].

Unituxin™ underwent priority review by the FDA and received approval with an orphan drug designation in 2015 to be used in combination with granulocyte-macrophage colony stimulating factor (GM-CSF), interleukin-2 (IL-2) and 13-cis-retinoic acid (RA), for the treatment of pediatric patients with high-risk neuroblastoma who achieve at least a partial response to prior first-line multiagent, multimodality therapy [55, 57].

### Platelet-derived growth factor receptor alpha (PDGFR-α)

PDGFR-α is a tyrosine kinase receptor expressed on cells of mesenchymal origin that plays a key role in gastrulation, central nervous system, gonads, lung, intestine, skin and skeleton [58]. The aberrant activation of the receptor has been detected on several human cancers including sarcoma and also stromal cells where signaling can contribute to the maintenance of the tumor microenvironment [59, 60].

#### Olaratumab (Lartruvo)

Olaratumab is a recombinant human IgG1 monoclonal blocking antibody that binds specifically to human PDGFR-α preventing its binding with PDGF-AA and -BB ligands avoiding the activation of the receptor and downstream signaling [60].

Lartruvo™ is approved under accelerated approval in combination with doxorubicin for the treatment of adult patients with soft tissue sarcoma (STS) which is not amenable to curative treatment with radiotherapy or surgery but candidate for an anthracycline-containing regimen [60].

## Blockade of angiogenesis

Angiogenesis occurs during development and vascular remodeling as a controlled process that leads to neovascularization where the blood vessels and stromal components are responsive to pro- and anti-angiogenic factors [61]. However, in pathological situations such as cancer, it is universally recognized that tumor cells can induce the angiogenic pathway leading to the neo-angiogenesis, a process that is associated with tumor progression and poor prognosis in cancer patients [61, 62]. Therefore, as angiogenesis is essential for tumor growth and metastasis, controlling tumor-associated angiogenesis became a promising strategy as an anticancer therapy.

### VEGF/VEGFR

Since VEGF is one of the most important mediators of neo-angiogenesis and tumor growth [4], the blockade of VEGF is the most relevant example of this group. There are five members of the VEGF family that have been described to date: VEGF-A, VEGF-B, VEGF-C, VEGF-D and VEGF-E. From all these, VEGF-A is considered to be the most important and its functions are mediated by the interaction either with VEGF receptor 1 or 2 (VEGFR-1 and VEGFR-2, respectively) [4]. The importance of VEGF-A and VEGFR-2 in tumor angiogenesis suggests that blockade of this ligand and receptor could be a useful therapeutic strategy for inhibiting angiogenesis and tumor growth. For that reason VEGF-A and VEGFR-2 become the main targets of current antiangiogenic agents. Interesting, VEGF also elicits epithelial-mesenchymal transition via an autocrine loop [63], suggesting that it is also involved in the early propagation of malignant cells outside the epithelial layer. Below, we described the anti-angiogenesis mAbs approved for clinical use.

#### Bevacizumab (Avastin®)

Bevacizumab is a recombinant humanized monoclonal IgG1 antibody that binds to all isoforms of VEGF-A preventing the interaction with its receptors and their subsequent activation [64]. The result is a regression of immature tumor vasculature, normalization of remaining tumor vasculature and inhibition of further tumor angiogenesis [65, 66]. In the last 10 years, bevacizumab has been studied for various diseases reaching many indications for solid cancers such as mCRC, metastatic breast cancer, metastatic renal cell carcinoma, metastatic ovarian cancer, advanced non-Small Cell Lung Cancer (NSCLC) and glioblastoma.

Avastin® was first approved by the FDA in 2004 as first-line treatment in mCRC, administered in combination with intravenous 5FU–based chemotherapy [67]. Later, its approval was extended as a second-line treatment of mCRC patients in addition to 5-fluorouracil, irinotecan or 5-fluorouracil, oxaliplatin based chemotherapy [68–70]. In 2006, Avastin® was approved as first-line in combination with carboplatin and paclitaxel in unresectable, locally advanced, recurrent or metastatic non-squamous non-small cell lung cancer [70, 71]. The use of Avastin® in combination with paclitaxel as first-line treatment in HER2-negative metastatic breast cancer was rapidly approved in 2008, based on a preliminary analysis of the Phase III E2100 trial [72]. However, this approval was then revoked by FDA in 2011 after AVADO and RIBBON-1 trial showed no benefit in terms of OS [73, 74]. Contrary to FDA, the European Medicines Agency (EMA) maintained its approval to be used in combination with paclitaxel and with capecitabine in patients for whom other chemotherapy options including taxanes or anthracyclines are not appropriate [75]. In 2009, the FDA approved the use of Avastin® in combination with interferon alfa (IFN-α) for the treatment of metastatic renal cell carcinoma [70, 76] and as a single agent to treat glioblastoma after progression following prior therapy. In 2013, the FDA approved Avastin® to be used in combination with fluoropyrimidine–irinotecan- or fluoropyrimidine–oxaliplatin-based chemotherapy for the treatment mCRC in those cases where disease has progressed during the first line treatment with a bevacizumab containing regimen [70]. Finally, in 2014 Avastin® received the FDA approval to treat persistent, recurrent, or metastatic cervical cancer in combination with paclitaxel and either cisplatin or topotecan [70]. Also, to treat platinum-resistant recurrent epithelial ovarian, fallopian tube, or primary peritoneal cancer in combination with paclitaxel, pegylated liposomal doxorubicin, or topotecan based on the AURELIA trial [77].

#### Ramucirumab (Ciramza®)

Ramucirumab is a recombinant human monoclonal IgG1 antibody that binds to the extracellular domain of VEGFR-2 inducing conformational changes that results in the blockade of this receptor [78, 79]. Preclinical models showed that ramucirumab might selectively bind to and inhibit the human VEGFR-2 with a much greater affinity than its natural ligands [80, 81].

Ciramza® was first approved by the FDA in 2014 as single agent for the treatment of advanced or metastatic gastric or gastroesophageal junction (GEJ) adenocarcinoma. In particular, in those patients with disease progression after prior treatment with fluoropyrimidine- or platinum-containing chemotherapy [82, 83]. In addition, it has been approved for the treatment of metastatic NSCLC in patients with disease progression after platinum-based chemotherapy in combination with docetaxel [82]. Then, in 2015 the FDA extended its approval for the treatment of mCRC in patients with disease progression on or after prior therapy with bevacizumab, oxaliplatin and a fluoropyrimidine in combination with FOLFIRI [82]. Finally, the use of ramucirumab has being trialed in hepatocellular carcinoma as a second-line treatment (REACH trial) and in HER2-negative advanced breast cancer in combination with docetaxel (ROSE/TRIO-125 trial). However, published data did not show improvement in survival [84] or clinical outcomes [85], respectively.

## Modulation of immune response

In 1970 Burnet showed that tumor cells were capable to induce immune responses [86]. Nevertheless, it has also been demonstrated that tumors can escape from this immune pressure [87]. Among the strategies used by tumors to counteract the effector anti-tumor immunity, the modulation of intrinsic immunoregulatory mechanisms must be mentioned [88]. The activation of these mechanisms, known as “immune checkpoints” (IC), leads to a state of functional exhaustion of tumor-specific effector T cells in the tumor site [89, 90]. For that reason, the blockade of these ICs is an excellent way to enhance the effectiveness of the anti-tumor immune response elicited in cancer patients.

### CTLA-4

CTLA-4 is a structural homolog of CD28 co-stimulatory molecule and shares functional affinity for the B7 family molecules CD80 and CD86 [91]. Nevertheless, CTLA-4 acts as a negative regulator of T-cells. As CTLA-4 has a greater avidity for B7 molecules than CD28, once it is expressed on the surface of activated T-cells, it dampens T-cell activity and proliferation as well as cytokine production [92–94]. Since it plays a critical role as immune checkpoint, it became a perfect target to overcome negative regulation of immune response.

#### Ipilimumab (Yervoy®)

Ipilimumab is a recombinant human monoclonal antibody that binds to CTLA-4 and blocks the interaction of CTLA-4 with its ligands, CD80/CD86. The blockade increases T-cell activation and proliferation, including the tumor infiltrating T-effector cells. Inhibition of CTLA-4 signaling can also reduce T-regulatory cell function, which may contribute to a general increase in T cell responsiveness, including the anti-tumor immune response [95, 96].

Yervoy® was first approved by FDA in 2011 for the treatment of unresectable or metastatic melanoma. Then, in 2015 the FDA extended its approval for the additional indication of adjuvant treatment of patients with cutaneous melanoma with pathologic involvement of regional lymph nodes of more than 1 mm who have undergone complete resection, including total lymphadenectomy. The approval was based on improvement in recurrence-free survival (RFS) in a randomized (1:1), double-blind, placebo-controlled trial in 951 patients with resected Stage IIIA (lymph node >1 mm), IIIB, and IIIC (with no in-transit metastases) histologically confirmed cutaneous melanoma [95].

### PD-1

PD-1/PD-L1 axis regulates the functionality of T cells, B cells, NK cells, monocytes and a subset of dendritic cells (DC) [97–103] via its interaction with the two known ligands, PD-L1 (B7-H1) and PD-L2 (B7-DC). Additionally, the fact that the engage of PD-L1 with PD-1 inhibits the proliferation and cytokine production by T cells lymphocytes [104, 105] and that the PD-L1 also expresses on a number of human cancers like urothelial, gastrointestinal, lung, breast, melanoma and ovarian cancer [102, 106–114] prompted to the development of different mAbs to target either the receptor or the ligand.

#### Nivolumab (Opdivo®)

Nivolumab is a human monoclonal antibody that binds to the PD-1 receptor and blocks its interaction with PD-L1 and PD-L2, releasing the immune response inhibition mediated by PD-1, including the anti-tumor immune response [115]. Combined nivolumab (anti-PD-1) and ipilimumab (anti-CTLA-4) mediated inhibition results in enhanced T-cell function that is greater than the effects of either antibody alone, and results in improved anti-tumor responses in metastatic melanoma [115].

Opdivo® was first approved by the FDA in 2014 for the treatment of patients with unresectable or metastatic melanoma and disease progression following ipilimumab and, if BRAF V600 mutation positive, a BRAF inhibitor. In 2015 the FDA extended its approval in four opportunities; on March, for the treatment of patients with metastatic squamous NSCLC with progression on or after platinum-based chemotherapy; on September, for the treatment of unresectable or metastatic melanoma in combination with ipilimumab in BRAF V600 wild-type patients; on October, for the treatment of patients with metastatic NSCLC with progression on or after platinum-based chemotherapy and on November, for the treatment of advanced renal cell carcinoma in patients who have received prior anti-angiogenic therapy. Finally, in 2016 the FDA granted an accelerated approval for the treatment of patients with classical Hodgkin lymphoma (cHL) that has relapsed or progressed after autologous hematopoietic stem cell transplantation (HSCT) and post-transplantation brentuximab vedotin and in November for recurrent or metastatic squamous cell carcinoma of the head and neck with disease progression on or after a platinum-based therapy [115].

#### Pembrolizumab (Keytruda®)

Pembrolizumab is a humanized monoclonal antibody that also binds to PD-1 receptor blocking its interaction with PD-L1 and PD-L2 ligands [116].

Keytruda® was first approved by the FDA in 2014 for the treatment of patients with unresectable or metastatic melanoma and disease progression following ipilimumab and, if BRAF V600 mutation positive, a BRAF inhibitor. Then, in 2015 Keytruda® obtained two additional approvals; on October, for the treatment of patients with metastatic NSCLC expressing PD-L1 with disease progression on or after platinum-containing chemotherapy and on December, for the treatment of patients with unresectable or metastatic melanoma. Finally, in October 2016 the FDA granted an accelerated approval based on tumor response rate and durability of response for the treatment of patients with recurrent or metastatic head and neck squamous cell carcinoma (HNSCC) with disease progression on or after platinum-containing chemotherapy [116].

#### Atezolizumab (Tecentriq™)

Atezolizumab is an Fc-engineered, humanized, monoclonal antibody that binds to PD-L1 and blocks its interactions with both PD-1 and B7.1 receptors. This releases the PD-L1/PD-1 mediated inhibition of the immune response, including activation of the anti-tumor immune response without inducing antibody-dependent cellular cytotoxicity [117].

Tecentriq™ was approved by the FDA in 2016 for the treatment of patients with locally advanced or metastatic urothelial carcinoma who have a disease progression during or following platinum-containing chemotherapy or have a disease progression within 12 months of neoadjuvant or adjuvant treatment with platinum-containing chemotherapy. This indication is approved under accelerated approval based on tumor response rate and duration of response. Continued approval for this indication may be contingent upon verification and description of clinical benefit in confirmatory trials. In October 2016 it was also approved for the treatment of patients with metastatic NSCLC who have disease progression during or following platinum-containing chemotherapy or FDA-approved therapy for EGFR or ALK genomic aberration [117].

## Regulation of osteoclast function

Solid tumors like breast or prostate cancer or haematological conditions like multiple myeloma frequently metastasize to the bone [118]. The interaction between tumor cells and the bone matrix provokes osteoclast activation and the consequent bone destruction [119–121]. When osteoclasts resorb bone they release growth factors stored in the bone matrix, which in turn activate tumor cell proliferation, creating a vicious cycle. Osteoclast maturation and activation are mediated by receptor activator of NF-kappa B ligand (RANKL) after it binds to its receptor RANK expressed at the surface of mature osteoclasts and osteoclast precursor [122–125]. This process is tightly regulated by osteoprotegerin (OPG), a secreted protein that acts as a soluble decoy receptor preventing the binding of RANKL to its receptor RANK and blocking its activation [126]. Targeting osteoclast activation to interrupt this vicious cycle may therefore be a promising target for mAb immunotherapy in advanced cancer disease.

### Denosumab (Xgeva®, Prolia®)

Denosumab is a human IgG2 monoclonal antibody with a high affinity and specificity for human RANKL. By binding to RANKL, in a manner similar to that of native OPG, denosumab prevents its interaction with RANK thus, reducing the differentiation, activity and survival of osteoclasts which in turns reduce the release of growth factors [127].

Xgeva® was approved by the FDA in 2013 for the prevention of skeletal-related events (SRE) in patients with bone metastases from solid tumors including skeletally mature adolescents with giant cell tumor of bone that is unresectable or where surgical resection is likely to result in severe morbidity [128].

*Prolia®* was approved by the FDA in 2010. Todays it is indicated as a treatment to increase bone mass in patients at high risk for fracture receiving androgen deprivation therapy (ADT) for non-metastatic prostate cancer or adjuvant aromatase inhibitor (AI) therapy for breast cancer [128].

## Antibody-drug conjugates (ADC)

The huge effort made to merge the positive features of cytotoxic drugs (CDs) with the specificity of mAbs led to the development of ADC which consist in a cytotoxic drug (CD) conjugated to a mAb through a chemical linker. The CD selected to develop the ADCs are typically potent and poorly tolerated when used as free agents. Nevertheless, when they are covalently attached to the antibody, the linker provides to the ADCs sufficient stability to remain intact in circulation and labile enough to be released after internalization [129]. This approach allows directing a high concentration of the CD to the tumor environment reducing its side-effects and also widing its therapeutic window [130, 131]. As a consequence of the promising results, to date there are over 40 ADCs in clinical trial [132] with different molecular targets in both, solid and haematological cancers [133].

### Ado-trastuzumab emtansine (Kadcyla®)

Ado-trastuzumab emtansine is a HER2-targeted ADC which contains the humanized IgG1 anti-HER2, trastuzumab, covalently linked to the microtubule inhibitory drug DM1 (a maytansine derivative) via the stable thioether linker MCC (4-[N-maleimidomethyl] cyclohexane-1-carboxylate). Ado-trastuzumab emtansine contains an average of 3.5 DM1 molecules per antibody [134, 135]. The antitumour action of this ADC is related not only to the well-known role of trastuzumab, but also to the inhibition of microtubular assembly induced by DM1 on HER2-overexpressing cells. Indeed, trastuzumab delivers DM1 to the targeted tumour cells, focusing its toxicity almost only on cancer cells [136]. Moreover, it seems that T-DM1 is internalized after binding cancer cells’ surface receptors [137].

Kadcyla® was approved by FDA in 2013 as a single agent for the treatment of patients with HER2-positive, metastatic breast cancer who previously received trastuzumab and a taxane, separately or in combination. Patients should have either received prior therapy for metastatic disease or developed disease recurrence during or within six months of completing adjuvant therapy [134].

## Conclusion

The considerable progress that has been made in the field of monoclonal antibodies in cancer treatment since the first FDA approval in 1997 led to the inclusion of many mAbs in the standard of care as first- and second- line therapy for a number of solid tumors. There is no doubt the huge progress made since Milstein and Köhler found in 1975 how to produce them in continuous cultures and further after its introduction in clinical practice. Recent advances in molecular biology and protein engineering allowed the production of chimeric, humanized and even human mAbs as novel tools to treat cancer. Moreover, chimeric antibodies facilitated the delivery of highly toxic anti-cancer drugs directly to the tumor microenvironment.

As reviewed, many mAbs have been approved by the FDA to treat different types of solid tumors and most of them were developed to recognize almost the same tumor targets like HER2, EGFR, VEGF/R, CTL4 and PD1-PD-L1 and less extensively GD2 and RANKL with the purpose to block oncogenic pathways and the formation of new blood vessels, to modulate the immune response against tumor cells and to regulate osteoclast function and deliver cytotoxic drugs to the tumor cells. Clinical trials showed that the use of mAbs may improve the overall survival in many cancerous conditions as single agent or in combination with standard chemotherapy and, with the exception of Bevacizumab that was withdrawn in 2011 for the treatment of metastatic breast cancer, the rest of them are still available in clinical practice.

However, considering the initial expectations we can say the success has been limited but there is still room for improvement like for example expanding the biomarkers scope. In this regard during the writing of this manuscript, Olaratumab, a human IgG1 monoclonal antibody that binds to PDGFRα was approved by the FDA to be used in combination with doxorubicin for the treatment of adult patients with soft tissue sarcoma. We can also mention Xilonix™ that could break as a member of the next generation of mAbs being the first-in-class true human antibody derived from real patients who possess natural immunity to certain diseases. Xilonix™ blocks the IL-1α produced by the body in response to tumor growth and it is being tested in an FDA fast tracked, pivotal phase 3 study for the treatment of metastatic colorectal cancer (NCT02138422, NCT01767857).

A slightly different approach is used with the Chimeric Antigen Receptor into T cells (CAR-T) where the goal is to produce an immune-mediated antitumor response through the ex vivo manipulation of T cells. The underlying concept is to link an extracellular ligand recognition domain to an intracellular signaling domain of the TCR complex in order to induce T cell activation upon antigen binding. CAR-T showed promising results in haematological malignancies and they are also being explored in solid tumors. Recent review can be found elsewhere [138].

Great advances and several mAbs have also been approved by the FDA for the treatment of haematological cancer since Rituximab appeared on the scene to target CD20. We can remark the anti-CD20 Ibritumumab, Tositumomab, Ofatumumab and Obinutuzumab; the anti-CD38 Daratumumab; the anti-CD52 Alemtuzumab; the anti-SLAMF7 Elotuzumab and the anti-CD19 and -CD3 bispecific antibody Blinatumumab [139–145]. Likewise, we cannot forget that mAbs like Nivolumab originally tested on solid tumors already got its approval in 2016 for the treatment of patients with classical HL [115].

Regarding biosimilars, we must point out it is impossible to make exact copies of biologics as they are large and complex molecules produced in living cells. Consequently, there could be small differences in their immunogenicity compared with its references that could modify the clinical response and toxicity profile. However, there are several development programs for Rituximab (NCT01701232, NCT02260804, NCT02268045), Trastuzumab (NCT02162667, NCT01901146, NCT02187744) and Bevacizumab (NCT01763645, NCT02364999, NCT01966003) trying to overcome these difficulties. If they finally got the FDA approval, provably in the near future, the current clinical landscape will radically change.

After analyzing the current landscape of mAbs, it is clear that we still do not have the envisioned magic bullet to treat cancer but is also true that along the time improvements have been done in the development of targeted therapies and also on the identification of specific tumor markers that can be targeted. With the current knowledge, the rapid advance of science and the arrival of biosimilars it is highly probable that this century give us the light to let behind the old fashion approach of treating cancer patients with unspecific chemotherapy to finally move forward to a more tailored approach based on predictive biomarkers not just for mAbs or adoptive cellular therapy but also for small molecules.

## Abbreviations

ADC: Antibody-drug conjugates
ADCC: Antibody-dependent cellular cytotoxicity
ALCL: Anaplastic large cell lymphoma
ALL: Acute lymphoblastic leukemia
ASCT: Autologous stem cell transplant
CD: Cytotoxic drugs
CDC: Complement-dependent cytotoxicity
DC: Dendritic cells
EGFR: Epidermal growth factor receptor
EGFR: Epidermal growth factor receptor
EMA: European Medicines Agency
FDA: Food and Drug Administration
HL: Hodgkin lymphoma
MA: Malignant ascites
mAbs: Monoclonal antibodies
mCRC: Metastatic colorectal cancer
MMAE: Monomethyl auristatin E
NK: Natural killer cells
NSCLC: Non-small cell lung cancer
sALCL: Systemic anaplastic large cell lymphoma
scFV: Single chain variable fragment
TAA: Tumor associated antigens
